# General commentary: 28-Day oral chronic toxicity study of arctigenin in rats

**DOI:** 10.3389/fphar.2025.1720821

**Published:** 2026-01-22

**Authors:** Michelle Carnazza, Nan Yang, Jan Geliebter, Raj Tiwari, Victor Garcia, Xiu-Min Li

**Affiliations:** 1 Division of R&D, General Nutraceutical Technology, LLC, Briarcliff Manor, NY, United States; 2 Department of Pathology, Microbiology and Immunology, New York Medical College, Valhalla, NY, United States; 3 Department of Otolaryngology, New York Medical College, Valhalla, NY, United States; 4 Department of Pharmacology, New York Medical College, Valhalla, NY, United States; 5 Department of Dermatology, New York Medical College, Valhalla, NY, United States

**Keywords:** toxicology, arctigenin, pharmacology, pharmacokinectics, dose response

Arctigenin is a bioactive constituent of burdock (Arctium lappa), a plant widely consumed as a vegetable in China, Japan, and Korea as the authors note. The root of burdock is broadly utilized in culinary applications and is also recognized for medicinal properties, including gastrointestinal benefits and antioxidant activity (Yosri et al., 2023). Notably, arctigenin belongs to the polyphenol class of compounds, which are generally in fruits and vegetables and are regarded as safe and associated with diverse health-promoting effects (Duda-Chodak and Tarko, 2023).

In the study by Tan et al. (2018), the authors tested three daily doses of arctigenin (ARC), at 12 mg/kg (1x/minimal dose), 36 mg/kg (3x dose), and 120 mg/kg (10x dose). The treatments consisted of 28 days of feeding in 3 individual groups, respectively, plus a vehicle control group, followed by 28 weeks of recovery. Safety endpoints included body weight, food intake, urine and blood biochemistry, histopathology, and plasma concentration (AUC).

The strengths of this study include its study design, which incorporates both male and female rats considering sex as a biological variable, the dosing regimen/protocol including the sub-chronic exposure studies, as well as the detailed description of the results and comprehensive discussion in the body part of the manuscript. However, we realized some inaccuracy throughout the description of results and, importantly, in the abstract. Specifically, the authors did not include a key finding wherein the high dose (3x (36 mg/kg) and 10x (120 mg/kg) dose of ARC) arms did not cause histopathological abnormalities, but the minimal dose (1x (12 mg/kg) dose of ARC) caused abnormalities that matched what was observed in the vehicle/placebo control group, an intrinsic pathology inherent to the rat model used.

The results indicated no significant differences in body weight or food intake between treatment groups, across both sexes. Although the authors reported “a significant reduction in body weight in the high dose group”, the corresponding figure (Figure 1A-B) showed overlapping lines across groups with a lack of statistics. Furthermore, the placebo (vehicle) group showed a marked reduction in food intake on Day 14 (Figure 1C), which was not addressed in the Results section nor Discussion section. The abstract reads “The high dosage of *Arctigenin* only decreased the body weight at day 4”. Again, the body weight showed no difference between the three artigenin doses and the vehicle controls on day 4, as shown in Figure 1. Clear statistical analysis of these days/time-points would benefit the study and allow for a more robust interpretation of results.

Additionally, urine, hematological and blood biochemistry, and electrolyte parameters did not differ significantly with ARC treatment. Histopathological abnormalities in various organs were only observed in the minimal dose group (12 mg/kg), while the 3x and 10x ARC dose groups were histologically normal. Importantly, the AUC study showed that the 10x dose group exhibited 2 to 5 times higher blood concentration of ARC than the 3x ARC treatment group, indicating high exposure; however, both 3x and 12x ARC groups of rats exhibited an absence of pathological abnormalities. A toxicokinetic study (Figure 4 and Table 9) was not shown for the 1x ARC treatment group that exhibited pathological abnormalities.

The authors acknowledged and adequately discussed their results, that the abnormalities were not presented in the high-dose ARC groups, but in the minimal dose ARC group. In their discussion, the authors suggested that the lack of dose-related pathological changes might be due to an intrinsic pathology of the rats, as the vehicle/placebo group exhibits the same “pathology” as the minimal dose group. Indeed, several “representative” images of abnormalities were derived from the placebo group (Figures 2B, 2C, 2D, 2F, 2G, 2H, 3A, 3B, 3G). However, these results were not accurately described in the abstract.

We would like to emphasize the importance of including a stronger and more comprehensive summary of the results of the study throughout the body of the abstract. The abstract serves as the primary lens through which busy clinicians, researchers and consumers engage with a study, consequently, its clarity and precision are paramount, as it largely determines the perceived significance and credibility of the work. In its current form, the abstract, the authors conclude that the minimal adverse effect dose is 12 mg/kg in the absence of acknowledging the observation that higher doses, including 3x and 10X ARC doses, did not produce adverse effects and, in fact, were histologically normal. Therefore, the omission of these results from the abstract may suggest and influence readers to perceive that the 12 mg/kg is the lowest dose causing adverse effects. A more appropriate approach would be to conduct experiments in a model that does not have intrinsic pathologies that are commonly observed during vehicle/placebo interventions to avoid confounding factors. The study by Tan et al. (2018) should not include 12 mg/kg of arctigenin as the lowest observed adverse effect level (LOAEL) because their higher doses of arctigenin had no toxicity. Further studies are needed to define an optimal dose range of arctigenin and genereate no observed adverse effect level (NOAEL) and LOAEL.

We thank the authors for their contribution and the opportunity to engage in scholarly dialogue, and we respectfully suggest that inclusion of these points in the abstract would more accurately reflect the data presented.
